# Prediction of Early Diagnosis in Ovarian Cancer Patients Using Machine Learning Approaches with Boruta and Advanced Feature Selection

**DOI:** 10.3390/life15040594

**Published:** 2025-04-03

**Authors:** Tuğçe Öznacar, Tunç Güler

**Affiliations:** 1 Department of Biostatistics, Ankara Medipol University, Ankara 06570, Turkey; 2 Department of Medical Oncology, Park Hayat Hospital, Afyonkarahisar 03100, Turkey; tuncetto74@gmail.com

**Keywords:** feature selection, machine learning, Boruta, recursive feature elimination, CatBoost

## Abstract

Objectives: Ovarian cancer continues to be one of the most prevalent gynecological cancers diagnosed. Early detection is highly critical for increasing survival chances. This research aims to assess the feature extraction process from various machine learning techniques for better modelling of ovarian cancer and the selection process in ovarian cancer analysis. By eliminating irrelevant features, this approach could guide clinicians towards more accurate results and optimize diagnostic precision. Methods: This study included both patients with and without ovarian cancer, creating a dataset containing 50 independent variables/features. Eight machine learning algorithms: Random Forest, XGBoost, CatBoost, Decision Tree, K-Nearest Neighbors, Naive Bayes, Gradient Boosting, and Support Vector Machine, were evaluated alongside four feature selection techniques: Boruta, PCA, RFE, and MI. Metrics performance has been evaluated to obtain the best possible combination for diagnosis. Results: These results were obtained using these methods with a significantly reduced number of features. Random Forest and CatBoost’s performances demonstrated significant differences in contrast to other algorithms (respectively, AUC 0.94% and 0.95%). On the other hand, feature selection methods such as Boruta and RFE consistently reflected higher AUC and accuracy scores than the others. Conclusions: This study highlights the importance of choosing appropriate machine learning algorithms and feature selection techniques for ovarian cancer diagnosis. Boruta and RFE showed high accuracy. By reducing the number of features from 50 to the most relevant ones, clinicians can make more precise diagnoses, enhance patient outcomes, and reduce unnecessary tests.

## 1. Introduction

Ovarian cancer is diagnosed in approximately 300,000 women globally each year, making it the seventh most common cancer among women, according to GLOBOCAN [1]. Siegel et al. noted that in over 70% of patients, the disease is diagnosed in its late stages; hence, the survival rate drops to 30–50% within five years [2]. Ovarian cancer is the second most common gynecologic malignancy in resource-rich countries and the third most common in resource-limited countries, where cervical cancer is the most prevalent [3]. The majority of ovarian malignancies (95%) are epithelial, while the remaining cases arise from other ovarian cell types, such as germ cell tumors and sex cord-stromal tumors. In the United States, ovarian cancer is the second most common gynecologic malignancy and the leading cause of gynecologic cancer-related deaths. The average age at diagnosis is 63 years [4], and the lifetime risk of developing ovarian cancer is 1.3%.

Epithelial ovarian cancer is a histologic diagnosis based on pathological evaluation of tissue obtained after surgical removal of an ovary or fallopian tube, or through biopsies of the peritoneum. Less frequently, the diagnosis is established using tissue or fluid samples obtained via image-guided biopsy, paracentesis, or thoracentesis, all of which are standard diagnostic procedures. Early detection of ovarian cancer significantly improves prognosis, with survival rates reaching up to 90% when appropriate measures are taken [5]. However, a major challenge for both patients and medical professionals is the lack of adequate imaging technologies and the difficulty in accurately differentiating between various imaging modalities [6].

In recent years, there has been a growing interest in the application of machine learning and artificial intelligence in biomedical data analysis, including ovarian cancer research [7]. Several studies have explored the use of these algorithms for diagnosis and prognosis, demonstrating promising results [8]. However, despite these advancements, significant challenges remain in achieving clinically reliable and interpretable models for ovarian cancer diagnosis [9]. Further research is needed to assess the clinical applicability of these algorithms and their integration into routine practice. Beyond assisting in diagnosis, incorporating machine learning into clinical workflows has the potential to enhance clinicians’ decision-making capabilities, leveraging the sophisticated neural networks integrated within these algorithms [10].

The purpose of this research is to create machine-based learning models capable of diagnosing ovarian cancer at the earliest stages and then compare the models with respect to their diagnostic capabilities. In addition, considering the high dimensional nature of the datasets often encountered in this domain, this study aims to implement and compare the most commonly used feature selection methods in order to identify and utilize the most relevant features. The enhancement of algorithmic performance and the ability to make more useful clinical interpretations are sought through effective feature selection techniques. This research seeks to address the issue of how ovarian cancer can be diagnosed using models built with artificial intelligence, thus achieving higher diagnostic precision and helping with personalized medicine.

## 2. Materials and Methods

The major aim of the research is to apply different machine learning approaches in the construction of strong predictive models for the diagnosis of ovarian cancer. The dataset consists of 50 features collected from 349 patients, including 171 patients without ovarian cancer and 178 patients diagnosed with ovarian cancer. All the variables initially included in the model are described Table 1.

In order to do this, a comprehensive set of approaches was applied in sequence, which included data preprocessing, input feature selection, and dimensionality reduction. The methodology is outlined as follows:

### 2.1. Data Preprocessing

As the first step, an extensive data cleaning process of the dataset was carried out. This was done to facilitate data standardization. Missing values in the dataset were handled using IterativeImputer, a statistical method designed to impute missing values based on the relationships between the features in the dataset. IterativeImputer predicts each missing value iteratively by modeling it as a function of the other features. This process is repeated for a predefined number of iterations, which, in this case, was set to 10 as per the default configuration. The use of IterativeImputer was chosen due to its ability to provide more accurate imputations, particularly when there are strong correlations between the features [11,12].

### 2.2. Feature Selection and Dimensionality Reduction

To enhance model performance, feature selection and dimensionality reduction were carried out, focusing on minimizing overfitting and ensuring the model remained interpretable [13,14]. A number of techniques were used to identify the most applicable features for analysis:

Principal Component Analysis (PCA): PCA was utilized in decreasing the number of variables used whilst ensuring the information in the data maintained its relevance. The use of principal components in place of the original attributes helped in making the data representation less complicated, hence improving model performance. A variance threshold of 90% was set to assist with mitigating elements of complexity [15].

Boruta Algorithm: In essence, Boruta implements a feature selection process which involves comparing variables otherwise known as shadow features that are random. Any feature became irrelevant if it was significantly more important than the shadow version, which guarantees the relevancy of all the selected features [16].

Recursive Feature Elimination (RFE): RFE finds the maximum feature set by progressively deleting the features which ranked least important in the model. This approach was worth using due to its reliability in providing a credible feature importance score when utilizing the Random Forest method [17].

Mutual Information (MI): MI is a measure of the amount of information one variable contains about another. It quantifies the dependency between two variables by assessing how much knowing one variable reduces uncertainty about the other. MI is particularly useful for feature selection in machine learning, as it captures both linear and non-linear relationships. Higher mutual information between a feature and the target indicates that the feature provides valuable information for prediction [18].

### 2.3. Data Splitting

The dataset was split into training and testing sets, with 70% of the data used for training, and 30% for testing the model.

### 2.4. Classification Algorithms

This specific variety of algorithms was used on both the preprocessed and feature-selected datasets in isolation.

Random Forest: This is a form of ensemble where many decision trees are built and results are averaged for accuracy, bias, and overfitting purposes. In addition, it is also good for large datasets with a strong interaction between features [19].

XGBoost: This is a gradient boosting method, which is particularly popular, as it is quick and accurate. Simply, sequential trees are created by XGBoost in an attempt to overcome errors from other trees. This makes XGBoost effective in working with large datasets and allowing for model performance to improve through regularization [20].

CatBoost: CatBoost is a gradient boosting algorithm designed for categorical features, efficiently handling them without extensive preprocessing. It uses ordered boosting to reduce overfitting, making it effective for complex datasets with both numerical and categorical features [21].

Decision Tree: A model that is built using easily understandable rules that classify by splitting a feature space, designed to be used for easier understanding [22].

K-Nearest Neighbors (KNN): K-Nearest Neighbors is an algorithm that is nonparametric and distance-based. It classifies the instances according to the most frequent class of k nearest neighbor to them. Its easy to use but can become expensive in terms of computation when high dimensional data are involved [23].

Naive Bayes: A probabilistic model which is a type of classifier based on Bayes’s theorem with an assumption of independence among predictors. Although simple, it is efficient, data-based, and works nicely with small datasets [24].

Gradient Boosting: Gradient Boosting is a set of multiple basic models combined in a way that forms a single robust predictive model. In this kind of approach, every new model created is fitted to the errors made by the previous one, hence increasing the overall model performance [25].

Support Vector Machine (SVM): SVM is considered an effective tool for solving classification and regression problems. The goal is to determine the hyperplane that divides different classes of data points optimally [26].

Artificial Neural Network (ANN): ANNs are computational models inspired by the human brain’s structure and functioning. They consist of interconnected layers of neurons that process input data, learn from it, and make predictions. Through training, ANNs adjust their internal parameters to minimize errors, making them powerful tools for tasks such as classification, regression, and pattern recognition [27].

### 2.5. Hyperparameter Tuning

A hyperparameter tuning method called GridSearchCV was used on the dataset to help fine-tune it and help maximize the effectiveness of the various types of algorithms. This type of research is referred to as exhaustive because it searches through a specified hyperparameter grid to identify optimal combinations of hyperparameters [28,29,30,31]. The models were evaluated via a 5-fold cross validation architecture to ensure that overfitting was minimized and the results good and meaningful without being biased towards any one unique data split. The optimal hyperparameters for each model were selected based on the grid search results to maximize performance:

Random Forest: n_estimators = 200, max_depth = 10, min_samples_split = 5

XGBoost: n_estimators = 200, learning_rate = 0.1, max_depth = 6

CatBoost: iterations = 200, learning_rate = 0.1, depth = 10

Decision Tree: max_depth = 25, min_samples_split = 5

K-Nearest Neighbors: n_neighbors = 5, weights = ’uniform’

Gradient Boosting: n_estimators = 200, learning_rate = 0.1, max_depth = 6

SVM: C = 10, kernel = ’rbf’

ANN: hidden_layer_sizes = (100,) activation = ’relu’ solver = ’adam’ alpha = 0.0001 batch_size = ’auto’ learning_rate = ’constant’ learning_rate_init = 0.001 max_iter = 200 random_state = 42.

### 2.6. Evaluation Metrics

Several metrics were employed in determining the effectiveness with which the various models were able to work in prediction. Some of these metrics are described below.

The abbreviations used in the equations are as follows: TP: True Positive, TN: True Negative, FP: False Positive, FN: False Negative.

- Accuracy: The total number of correctly identified instances divided by the total population number [32,33,34].

(1) $$Accuracy=\frac{TP+TN}{TP+TN+FP+FN}$$

- Precision: This is the ratio of true positives out of all positive predictions, expressing the precision of the algorithm [32,33,34].

(2) $$Precision=\frac{TP}{TP+FP}$$

- Recall: In contrast to precision, recall is the ratio of true positives to the total number of actual positives, in other words, the sensitivity of the algorithm [32,33,34].

(3) $$Recall=\frac{TP}{TP+FN}$$

- F1 Score: F1 score is used to measure performance of the model and is the average of precision and recall [32,33,34].

(4) $$F1=\frac{2\;*\;Precision\;*\;Recall}{Precision+Recall}$$

- AUC (Area Under the Curve): This index assesses the ability of a model to perform discrimination between classes and has particular significance for unbalanced datasets [35].

**Figure 1:**
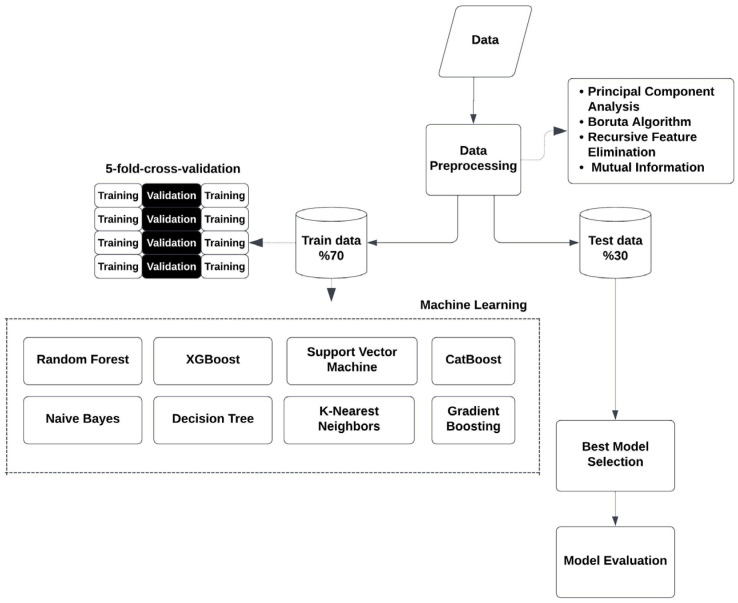
Flow Chart.

All implementations were conducted using Python (version 3.9.6) in Visual Studio Code (version 1.95.3). The following libraries and their versions were used: scikit-learn (1.5.2) for PCA, RFE, Mutual Information calculations, as well as for implementing Random Forest, Decision Tree, K-Nearest Neighbors, Naive Bayes, Gradient Boosting, and Support Vector Machine. BorutaPy (0.4.3) was used for the Boruta feature selection algorithm, while XGBoost (1.7.1) and CatBoost (1.2.0) were used for implementing the XGBoost and CatBoost algorithms, respectively. The entire process is illustrated in Figure 1.

## 3. Results

The results of this study provide a comprehensive evaluation of the performance of various machine learning algorithms and feature selection methods in predicting ovarian cancer, highlighting the effectiveness of different model and feature selection combinations. Algorithm performance has been tabulated in Table 2.

While predicting ovarian cancer, AFP, Age, ALB, ALP, AST, CA125, CA19-9, CA72-4, CEA, GLO, HE4, IBIL, LYM#, LYM%, MCH, Na, NEU, PCT, PLT, TBIL, and TP were the key features selected using Boruta feature selection with a set of 20. While Random Forest came second and achieved an accuracy of 86.67%, F1 86.67%, precision 86.89%, and AUC of 94.26%, Catboost outperformed all other algorithms with the highest performance rate of 89.52% accuracy, 89.46% F1, 90.73% precision, 89.52% recall, and 95.03% AUC. Even though XGBoost achieved an accuracy of 83.81%, F1 of 83.75%, 84.44% precision, 83.81% recall, and 92.26% AUC, it still underperformed compared to the Catboost and Random Forest algorithms in terms of precision and recall. Out of all, decision tree, KNN, Naive Bayes, Gradient boosting, and SVM algorithms performed the lowest, with a decision tree accuracy of 79.05% and AUC of 78.95%.

**Figure 2:**
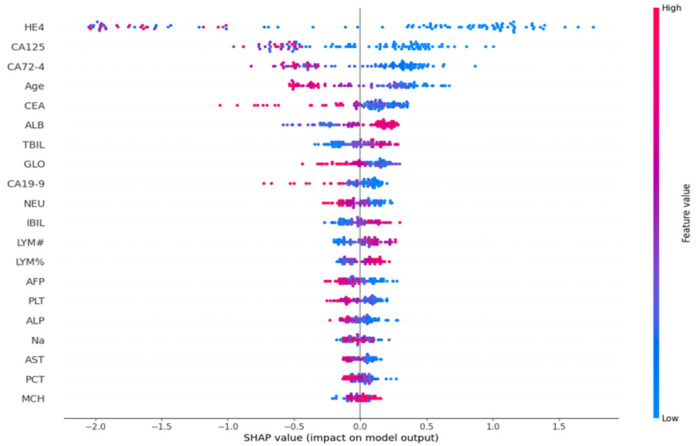
Summary Plot Using Boruta for Feature Selection and CatBoost for Modeling.

**Figure 3:**
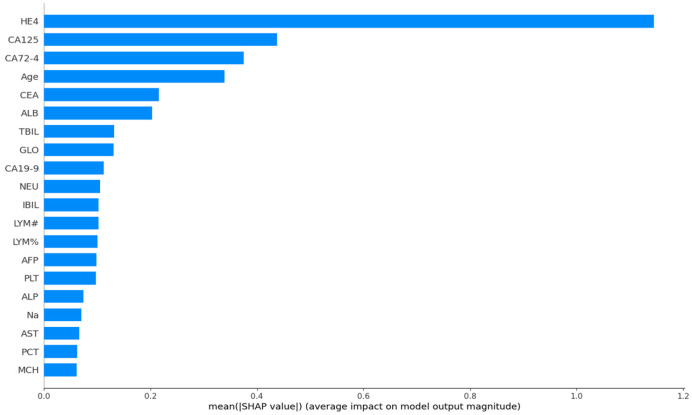
Feature Importance Bar Chart Using Boruta for Feature Selection and CatBoost for Modeling.

Figure 2 and Figure 3 illustrate the presented graphs, demonstrating the impact and relative importance of various features utilized in the diagnosis of ovarian cancer through SHAP value analysis. The results indicate that among all evaluated features, the biomarkers HE4, CA125, and CA72-4 exhibited the most significant influence on the model’s predictive outcomes.

**Figure 4:**
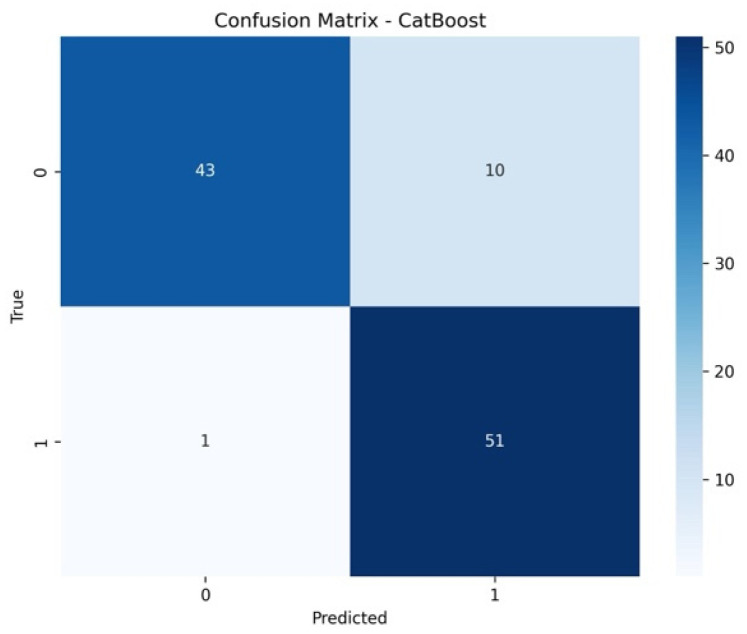
Confusion Matrix Using Boruta for Feature Selection and CatBoost.

The confusion matrix results indicate that the CatBoost model correctly classified 43 instances as class 0. However, the model misclassified 10 instances of class 0 as class 1. Similarly, only one instance of class 1 was incorrectly predicted as class 0. Lastly, the model successfully identified 51 instances of class 1, demonstrating strong predictive performance for the positive class. The model’s strength in differentiating between ovarian cancer and nonovarian cancer was clearly substantiated by the confusion matrix analysis. This balance between sensitivity and specificity further highlights the model’s expected future reliable clinical usefulness (Figure 4).

PCA analyzed the contribution of each feature, providing the weights (coefficients) that highlighted the most influential variables in the model, further enhancing the feature set by reducing noise and complexity. Under the usual performance measures, SVM proved to be the best model, boasting an outstanding performance of approximately 80% accuracy, 79.99% F1 score, and an AUC of 85.34%, all after PCA-based feature selection, which ensured dimensionality reduction. Both CatBoost and XGBoost achieved remarkable results, which were evident from their high accuracy and AUC values. Random Forest also demonstrated strong performance, yielding results that were comparable to those of other models. However, Decision Tree and Naive Bayes showed poor performance due to their significantly lower AUC values. The applications of SVM, CatBoost, and XGBoost in general PCA proved beneficial for overall model performance.

Applying RFE for feature selection, it was discovered that the key features for classification included AFP, Age, ALB, CA125, CA72-4, CEA, GLO, HE4, IBIL, and PLT. Random Forest proved to be the top performer under this criterion, with a total accuracy of 89.52%, an F1 Score of 89.52%, and AUC of 0.9505. Following closely was XGBoost, with accuracy and AUC of 83.81% and 0.9508, respectively, which helped place XGBoost among the top models. CatBoost showcased strong performance metrics as well and secured an accuracy of 88.57% AUC of 0.9430.

Using Mutual Information as a criterion for feature selection, a total of 10 features were chosen. These included HE4, CA125, CA72-4, Age, ALB, LYM%, CEA, Na, PDW, and AFP. The leaderboard was topped by CatBoost with an accuracy of 88.57%, F1 score of 88.54%, precision at 89.09%, recall at 88.57% and AUC at 92.96%. Following CatBoost was Random Forest, SVM, Naive Bayes, Gradient Boosting, XGBoost, K-Nearest Neighbors, and Decision Tree, from highest to lowest performance.

The performance of the ANN model was evaluated using various feature selection methods. The Boruta method achieved the highest accuracy at 84.76% and AUC at 88.28%, demonstrating its superior ability to identify relevant features. Although dimensionality reduction via PCA reduced the accuracy to 79.05%, it resulted in a slight increase in AUC, to 88.42%. Both RFE and Mutual Information methods produced comparable results, with Mutual Information showing a notable improvement in precision at 81.15% and recall at 80.95%, indicating its effectiveness in preserving the model’s discriminative power.

**Figure 5:**
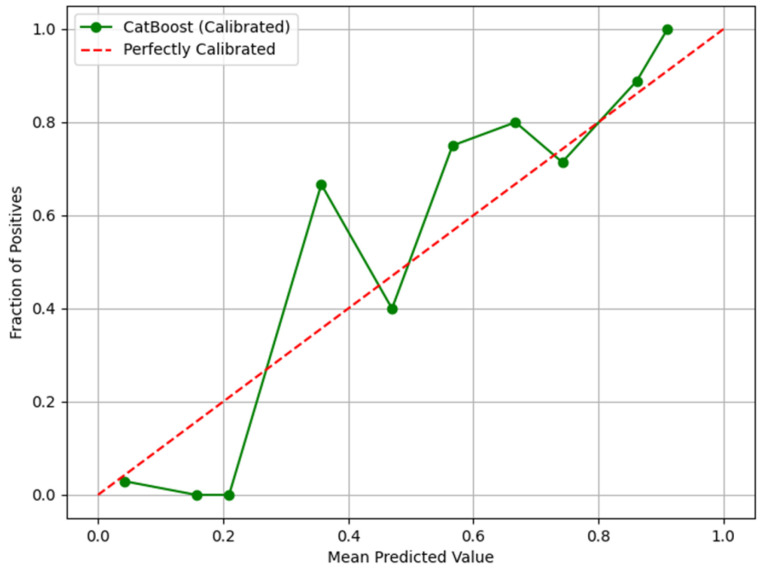
Calibration Curve Using Boruta for Feature Selection and CatBoost.

In Figure 5, the calibration curve of the CatBoost model demonstrates a generally consistent trend with the perfectly calibrated reference line, indicating a reasonable alignment between predicted probabilities and actual outcomes.

## 4. Discussion

This research studies the application of different ML approaches and feature extraction techniques in ovarian cancer prediction. From the results, it could be observed that more advanced models like Random Forest, XGBoost, and CatBoost improved their performance considerably with the integration of suitable feature selection methods, especially RFE and Boruta. The application of PCA and Mutual Information, however, resulted in a drop in the model’s performance. On the other hand, simpler models generally achieved lower accuracy. With the results, it is clear that placing importance on the right selection of feature selection methods, RFE and Boruta, for an optimal prediction is necessary. In addition, it is reported in this study that with the right feature selection methods, XGBoost and CatBoost perform the best, thereby enhancing the existing theories on the usage of ML techniques for prediction of ovarian cancer with advanced feature selection optimization.

Although hybrid methods using stacking and voting techniques were also tested in this study, no significant improvement in performance was achieved. Additionally, to further assess the model’s generalization capacity, experiments were conducted using a dataset split into training, validation, and test subsets. The results demonstrated that the performance on the validation set was consistent with that of the test set, with the CatBoost algorithm achieving higher performance during the validation phase.

According to the findings of Li-Rong Yang et al., in their research, they delved into predicting platinum-resistant recurrence in epithelial ovarian cancer, and their findings were impressive as well, thanks to the XG Boost model, which was formed from variables selected via multiple logistic regression. This model was able to achieve an AUC of 0.784, a sensitivity of 0.735, specificity of 0.713, and an accuracy of 80.4%. However, in the study that we conducted, we found that the CatBoost model that made use of Boruta feature selection was most prominent, with a combination of AUC of 0.950 and accuracy of 89.5%. The data used for the model were laboratory and clinical data [36].

Using 33 blood features and radical machine learning, Xiaopei Chao et al. managed to construct a blood risk score that led to a C-index value of 0711 and was able to outperform the AUC with regard to conventional factors. In contrast, our research is concerned with improving methods of ovarian cancer prediction through the optimization of feature selection techniques [37].

Seyed Mohammad Ayyoubzadeh-al et al., in their research, were able to enhance its accuracy up to 86% while predicting ovarian cancer. This was achieved using artificial neural networks and SVM, to name a few. The set that was used is quite similar to the one we had. In contrast, it is evident that our model performed better, as we managed to obtain accuracy of 89.5% and an AUC of 0.950 using Boruta (version 0.4.3) and Catboost (version 1.2.0), respectively. Such models showcase the strength of feature selection techniques when predicting ovarian cancer [9].

The same ovarian cancer dataset was utilized by Sheela Lavanya JM and Subbulakshmi P, who employed SVM and ensemble learning techniques through stacking, along with Minimum Redundancy Maximum Relevance, for feature selection. Their model achieved 89% accuracy. In our study, we explored additional feature selection techniques, including Boruta, PCA, RFE, and Mutual Information, alongside various machine learning algorithms. This approach resulted in a slight improvement in performance, with the CatBoost model achieving accuracy of 89.5% and an AUC of 0.950. These results suggest that our feature selection methods may contribute to improved prediction of ovarian cancer [38].

Amniouel et al. examined the task of finding gene biomarkers for chemotherapy response in serous ovarian cancer (SOC), using feature selection (LASSO and varSelRF) and machine learning classifiers for achieving high prediction accuracy. On the other hand, the current study sought to measure the performance of machine learning algorithms for feature selection for early diagnosis in an effort to reduce the features and increase the clinical relevance of the diagnosis. Both studies address the crucial component of feature selection in model performance improvement [39].

In Paik et al.’s study to predict survival outcomes of epithelial ovarian cancer, the authors noted AUCs of 0.830 and 0.843 for the training and validation cohorts, respectively, using gradient boosting. The model showed better performance as compared to the traditional Cox proportional hazard regression model, which had much lower AUCs. Similarly, our study found that the CatBoost and Random Forest models also performed well, achieving AUCs of 0.94% and 0.95%, respectively [40].

Gui et al. developed a predictive model using machine learning for ovarian cancer diagnosis, which achieved accuracy of 88% with LightGBM. The model fused clinical data like laboratory tests and imaging reports, enabling diagnosis 17 days earlier than clinical pathological examination. In contrast, our work sought to apply machine learning using Random Forest (version 1.5.2) and CatBoost (version 1.2.0) at AUC values of 0.94% and 0.95%, respectively, while concentrating on feature selection to improve diagnostic accuracy [41].

Chen et al. introduced an explainable ML model that predicts PFS in patients with high-grade serous ovarian carcinoma. The model identified residual tumor, HE4, and CA125 as significant predictors. Their model scored a C-index of 0.755. In relation, and as part of our research, HE4 and CA125 were also important factors in the early diagnosis of ovarian cancer, where our model reached an AUC of 0.94% and 0.95%. All studies corroborate the relevance of these biomarkers for ovarian cancer diagnosis and prognosis [42].

Piedimonte et al. analyzed the use of ML and radiomics in ovarian cancer with special emphasis on predicting responses to treatment. They uncovered that Random Forest and Neural Networks performed well, achieving an accuracy rate of 93.7% in predicting progression-free survival after 12 months. Likewise, in the study, we employed Random Forest and CatBoost models with AUC values of 0.94% and 0.95%, respectively. While treatment response prediction was the emphasis of Piedimonte et al., our focus was on more advanced ML features concerning ovarian cancer and its early diagnosis [43].

A key challenge in our study is the computational cost associated with feature selection methods. The computational costs of these methods vary depending on the technique used and the size of the dataset. Methods such as Boruta and RFE are often computationally intensive, as they require multiple iterations over the data and retraining of models. Simpler methods, however, are generally faster but may offer less accurate results. Therefore, the choice of method involves a trade-off between accuracy and computational efficiency. Additionally, another challenge in our study is the relatively small dataset. While the current dataset provided valuable insights, a larger dataset would enable more robust model training and improve the generalizability of the findings. Future studies could benefit from incorporating a larger sample size, which would enhance the reliability of the results, facilitating the application of more complex models.

## 5. Conclusions

This research emphasizes the importance of particular feature selection methods in the construction of accurate ovarian cancer predictive models. The use of Boruta feature selection together with CatBoost modeling gave exceptional results, which proved the feasibility of utilizing a large number of features to achieve high accuracy. It is noteworthy that the combination of RFE and Random Forest achieved satisfactory performance, even with a relatively small number of features, highlighting the effectiveness of selective approaches. These results have important implications, for instance, the engineering of improved early diagnosis and treatment modalities for ovarian cancer, as they emphasize the important roles that advanced feature selection and machine learning methods have in predicting ovarian cancer. Future work should seek to increase datasets and add other clinical and molecular parameters in order to improve model accuracy and clinical usefulness even further.

## Figures and Tables

**Table 1:** Variables and Their Definitions.

Variable	Description
**AFP**	Alpha-fetoprotein; a tumor marker primarily used to assess liver function and detect certain cancers.
**AG**	Albumin/Globulin ratio; a diagnostic indicator of liver function and protein balance.
**Age**	The age of the individual, typically used as a demographic variable.
**ALB**	Albumin; a protein produced by the liver, used to assess liver function and nutritional status.
**ALP**	Alkaline phosphatase; an enzyme related to liver, bone, and bile duct function.
**ALT**	Alanine aminotransferase; an enzyme that helps assess liver damage.
**AST**	Aspartate aminotransferase; an enzyme that is indicative of liver and heart function.
**BASO#**	Absolute basophil count; basophils are a type of white blood cell involved in immune responses, including allergies.
**BASO%**	Percentage of basophils among total white blood cells.
**BUN**	Blood urea nitrogen; a marker used to evaluate kidney function.
**Ca**	Calcium; a mineral essential for bone health, muscle function, and nerve signaling.
**CA125**	Cancer antigen 125; a biomarker used to assess ovarian cancer.
**CA19-9**	Cancer antigen 19-9; a marker used to assess pancreatic cancer.
**CA72-4**	Cancer antigen 72-4; a tumor marker mainly used for gastric cancer.
**CEA**	Carcinoembryonic antigen; a protein often elevated in various cancers, particularly colorectal cancer.
**CL**	Chloride; an electrolyte that helps maintain fluid balance and acid–base status.
**CO2CP**	Carbon dioxide content; measures the blood’s bicarbonate concentration, important for assessing acid–base balance.
**CREA**	Creatinine; a waste product of muscle metabolism, commonly used to assess kidney function.
**DBIL**	Direct bilirubin; a form of bilirubin that is conjugated in the liver and used to assess liver function and jaundice.
**EO#**	Absolute eosinophil count; eosinophils are white blood cells involved in allergic responses and parasitic infections.
**EO%**	Percentage of eosinophils among total white blood cells.
**GGT**	Gamma-glutamyl transferase; an enzyme used to evaluate liver and biliary system disorders.
**GLO**	Globulin; a class of proteins that includes immunoglobulins, which play a role in immune function.
**GLU**	Glucose; a key source of energy for cells, its levels are used to assess metabolic function and diabetes.
**HCT**	Hematocrit; the proportion of blood that is composed of red blood cells, used to assess anemia or dehydration.
**HE4**	Human epididymis protein 4; a biomarker for ovarian cancer detection.
**HGB**	Hemoglobin; a protein in red blood cells that carries oxygen from the lungs to the tissues.
**IBIL**	Indirect bilirubin; the unconjugated form of bilirubin, elevated in liver dysfunction and hemolysis.
**K**	Potassium; an essential electrolyte that regulates cell function, heart rhythm, and muscle contractions.
**LYM#**	Absolute lymphocyte count; lymphocytes are a subset of white blood cells that are critical for immune function.
**LYM%**	Percentage of lymphocytes among total white blood cells.
**MCH**	Mean corpuscular hemoglobin; a measure of the average amount of hemoglobin per red blood cell.
**MCV**	Mean corpuscular volume; the average volume of a red blood cell, used to classify anemia.
**Mg**	Magnesium; a mineral important for muscle and nerve function and enzymatic processes.
**MONO#**	Absolute monocyte count; monocytes are white blood cells involved in immune response and inflammation.
**MONO%**	Percentage of monocytes among total white blood cells.
**MPV**	Mean platelet volume; a measure of the size of platelets in the blood, used to assess platelet production and function.
**Na**	Sodium; an electrolyte that helps regulate fluid balance, blood pressure, and nerve function.
**NEU**	Neutrophils; the most abundant type of white blood cell, important for fighting bacterial infections.
**PCT**	Procalcitonin; a biomarker used to detect bacterial infections and assess sepsis.
**PDW**	Platelet distribution width; a measure of the variability in platelet size, useful for assessing platelet function.
**PHOS**	Phosphate; a mineral important for bone health and cellular energy production.
**PLT**	Platelets; cells involved in blood clotting and wound healing.
**RBC**	Red blood cells; cells responsible for oxygen transport in the body.
**RDW**	Red cell distribution width; a measure of the variability in red blood cell size, useful for diagnosing anemia.
**TBIL**	Total bilirubin; a combination of direct and indirect bilirubin, used to assess liver function and jaundice.
**TP**	Total protein; the sum of albumin and globulin in the blood, reflecting overall nutritional and liver status.
**UA**	Uric acid; a waste product of purine metabolism; elevated levels can indicate kidney dysfunction or gout.
**Menopause**	A binary categorical variable indicating whether the individual is postmenopausal.
**TYPE**	A binary categorical variable.

**Table 2:** Performance Comparison of Machine Learning Models with Different Feature Selection Methods for Ovarian Cancer Prediction.

Model	Method	Accuracy	F1	Precision	Recall	AUC
Random Forest	Boruta	0.8667	0.8665	0.8689	0.8667	0.9426
	PCA	0.7238	0.7228	0.72821	0.7238	0.8146
	RFE	0.8952	0.8952	0.8954	0.8952	0.9505
	Mutual Information	0.8571	0.8569	0.8605	0.8571	0.9307
XGBoost	Boruta	0.8381	0.8375	0.8444	0.8381	0.9216
	PCA	0.7429	0.7424	0.7453	0.7429	0.8320
	RFE	0.8381	0.8378	0.84133	0.8381	0.9508
	Mutual Information	0.8381	0.8378	0.8413	0.8381	0.9175
**CatBoost**	**Boruta**	**0.8952**	**0.8945**	**0.9073**	**0.8952**	**0.9502**
	PCA	0.7524	0.7511	0.7587	0.7524	0.8396
	RFE	0.8857	0.8856	0.8881	0.8857	0.9430
	Mutual Information	0.8857	0.8854	0.8909	0.8857	0.9296
Decision Tree	Boruta	0.7905	0.7904	0.7909	0.7905	0.7896
	PCA	0.6190	0.6186	0.6200	0.6190	0.6540
	RFE	0.8285	0.8284	0.8289	0.8285	0.8494
	Mutual Information	0.7904	0.7902	0.7923	0.7904	0.7908
K-Nearest Neighbors	Boruta	0.8190	0.8190	0.8191	0.8190	0.8534
	PCA	0.7428	0.7378	0.7650	0.7428	0.7821
	RFE	0.8285	0.8276	0.8366	0.8285	0.8798
	Mutual Information	0.8285	0.8283	0.8306	0.8285	0.8844
Naive Bayes	Boruta	0.8000	0.7986	0.8093	0.8000	0.9023
	PCA	0.7142	0.7079	0.7369	0.7142	0.7634
	RFE	0.8190	0.8164	0.8402	0.8190	0.9296
	Mutual Information	0.838095	0.836465	0.853893	0.838095	0.929245
Gradient Boosting	Boruta	0.8476	0.8472	0.8523	0.8476	0.9183
	PCA	0.7333	0.7319	0.7392	0.7333	0.8004
	RFE	0.8571	0.8565	0.8637	0.8571	0.9346
	Mutual Information	0.8476	0.8472	0.8523	0.8476	0.9174
SVM	Boruta	0.8476	0.8474	0.8497	0.8476	0.8762
	PCA	0.8000	0.7998	0.8010	0.8000	0.8534
	RFE	0.8761	0.8759	0.8797	0.8761	0.9154
	Mutual Information	0.8761	0.8753	0.8877	0.8761	0.8925
ANN	Boruta	0.8476	0.8472	0.8524	0.8476	0.8828
	PCA	0.7905	0.7845	0.8297	0.7905	0.8842
	RFE	0.7905	0.7894	0.7977	0.7905	0.8157
	Mutual Information	0.8095	0.8093	0.8115	0.8095	0.8330

## Data Availability

The dataset is available at https://www.kaggle.com/datasets/saurabhshahane/predict-ovarian-cancer/data (accessed on 31 March 2025).

## Abbreviations

The following abbreviations are used in this manuscript:

PCA	Principal Component Analysis
RFE	Recursive Feature Elimination
MI	Mutual Information
SVM	Support Vector Machine
ANN	Artificial Neural Network
AUC	Area Under the Curve
TP	True Positive
TN	True Negative
FP	False Positive
FN	False Negative
